# ﻿Taxonomic identity of *Distapliastylifera* (Tunicata, Ascidiacea), a new arrival to the eastern Pacific displaying invasive behavior in the Gulf of California, Mexico

**DOI:** 10.3897/zookeys.1157.95986

**Published:** 2023-04-05

**Authors:** Betzabé Moreno-Dávila, Leonardo Huato-Soberanis, Jaime Gómez-Gutiérrez, Carolina Galván-Tirado, Carlos Sánchez, Teresa Alcoverro, Eduardo F. Balart, Xavier Turon

**Affiliations:** 1 Programa de Ecología Pesquera, Centro de Investigaciones Biológicas del Noroeste, C.P. 23096, La Paz, BCS, Mexico; 2 Departamento de Plancton y Ecología Marina, Centro Interdisciplinario de Ciencias Marinas, Instituto Politécnico Nacional, C.P. 23096, La Paz, BCS, Mexico; 3 CONACYT, Universidad Autónoma de Baja California Sur, La Paz, BCS, Mexico; 4 Departamento de Ciencias Marinas y Costeras, Universidad Autónoma de Baja California Sur, La Paz, BCS, Mexico; 5 Department of Marine Ecology, Centre for Advanced Studies of Blanes (CEAB-CSIC), Blanes, Spain

**Keywords:** Ascidian, Gulf of California, introduced species, mass mortality, taxonomy, tunicate

## Abstract

A colonial ascidian of the genus *Distaplia* caused a mass mortality of the pen shell *Atrinamaura* (Sowerby, 1835) during June 2016 in the southwest of the Gulf of California (Mexico), with a significant socio-economic cost. Tentatively identified in previous works as Distapliacf.stylifera, a precise taxonomic determination was still lacking. In the present work, based on a detailed morphological study, it is confirmed that this aggressive species is *Distapliastylifera* (Kowalevsky, 1874). Originally described from the Red Sea, the species currently has a wide circumtropical distribution (with the exception of the Eastern Pacific to date) and is reported as introduced in parts of its range. The present account thus represents an important range extension of this species. However, when revising the original description and later observations, the reported variability of several characters makes it likely that the binomen is in fact a complex of species, as is common in other ascidians with wide distributions. A complete morphological and genetic study including populations from the entire range of distribution would be necessary to settle the status of *D.stylifera*. Taxonomic uncertainties hinder a correct interpretation of biogeographical patterns and inference on the origin of the studied population. Nevertheless, the known introduction potential of the species, coupled with an explosive growth in an anthropized environment, and the lack of any previous reports in the Eastern Pacific, strongly suggest that the investigated population represents yet another instance of ascidian introduction. From the point of view of management, its invasive behavior is cause for great concern and warrants mitigation measures.

## ﻿Introduction

Taxonomic identification of introduced species is a pre-requisite of any meaningful study of their biology and ecology, including correct ecosystem management (Geller et al. 1997). However, the study of biological introductions in the sea is plagued with taxonomic issues, complicated by the decline of taxonomic expertise (Engel et al. 2021) and the failure to cope with global distributions common in introduced species.

Several species of ascidians, known as sea squirts, are highly successful invaders and cause significant damage, modification, or impact to their new habitats, displacing native species or causing harm in aquaculture farms in several regions of the world (Lambert 2007; Locke and Hanson 2011). However, ascidians are a difficult group from the point of view of taxonomy, with few specialists and scarce diagnostic characters that are also difficult to observe (Monniot et al. 1991). Ascidians with wide distributions have often been showcased as instances of cryptic speciation, whereby several genetic lineages have been lumped under the same nominal species such as *Cionaintestinalis* (Linnaeus, 1767) (Gissi et al. 2017), *Diplosomalisterianum* (Milne Edwards, 1841) (Pérez-Portela et al. 2013), *Styelacanopus* (Savigny, 1816) (Barros and Rocha 2020) and *Botryllusschlosseri* (Pallas, 1766) (Bock et al. 2012). Additionally, the conspecificity of populations of other widespread introduced ascidians has been supported by molecular evidence like *Styelaplicata* (Lesueur, 1823) (Barros et al. 2009; Pineda et al. 2011) and *Didemnumvexillum* Kott, 2002 (Stefaniak et al. 2009; Casso et al. 2019). Often, widely distributed species translocated to several areas of the world receive local names, and thus fail to be recognized as introduced, constituting instances of the so-called pseudo-indigenous species (Carlton 2009). Upon closer morphological and genetic examination, these species can be shown to correspond to taxa described elsewhere (e.g., Ordóñez et al. 2016; Viard et al. 2019). Detailed morphological observation, coupled whenever possible with genetic data and integration of biogeographic information, are necessary for a reliable identification of potentially introduced ascidian species.

Eleven introduced species of ascidians have been detected in Mexico and five of them in the Gulf of California (Bastida-Zavala et al. 2014). *Styelacanopus* was recorded at Estuario de Urías, Sinaloa during 2004 (Salgado-Barragán et al. 2004). Four introduced species of ascidians were recorded (*Botrylloidesviolaceus* Oka, 1927, *Botryllusschlosseri*, *Lissoclinumfragile* (Van Name, 1902) and *Polyclinumconstellatum* Savigny, 1816) several years later in various docks of Mazatlán, and an oyster farm located in Topolobampo, Sinaloa, Mexico (Tovar-Hernández et al. 2010, 2013). *Botrylloidesviolaceus* is the main species that grows on oyster cultures and natural seabeds in Atlantic Canada (Carver et al. 2006).

A rapid population growth of an ascidian, preliminarily identified as Distapliacf.stylifera, was detected in Ensenada de La Paz starting in June 2015 and causing one year later (June 2016) a mass mortality event on *Atrinamaura* (Sowerby, 1835), a bivalve whose natural populations are harvested and represents an economically relevant income for regional fishermen (NOS 2015; Moreno-Dávila et al. 2021). The abrupt and rapid colonization of the ascidian was facilitated by the abundant substrate provided by the pen shell, which reached mean densities in the area of more than 47 indvs/250 m^2^ (Moreno-Dávila et al. 2021). The pen shell fishery was closed since 2013 to allow the population to recover after years of over-exploitation, thus the rapid tunicate colonization was linked to the success in the recovery of the pen shell population, while other environmental variables (sea surface temperature, sea surface Chlorophyll-*a* concentration, and dissolved oxygen concentration) did not have a significant ecological influence (Moreno-Dávila et al. 2021). Results of a biofouling experiment carried out in Bahía de La Paz reported that Distapliacf.stylifera was the most abundant macro-organism in metal panels with sliding coatings based on silicone resins after the second month of the experiment (Galicia-Nicolas et al. 2018). Distapliacf.stylifera in Ensenada de La Paz is currently the basibiont of 28 epibiont polychaete species (Cardona-Gutiérrez 2021). These studies noted the increased abundance of Distapliacf.stylifera from 2015 to 2016. Its proliferation has thus caused relevant negative impacts, as it is the main biofouling organism in the area (Galicia-Nicolas et al. 2018), and positive impacts such as the availability of habitat for different species of polychaetes (Cardona-Gutiérrez 2021) that are likely to continue over time. However, so far there is no taxonomic work on *Distaplia* specimens collected in the Ensenada de La Paz to infer conclusively its taxonomic status and hence whether it can be a native or introduced species (Valéry et al. 2008, 2009; Warren 2021). Several previous ecological and pharmacological studies used the name *Distapliastylifera* (Kowalevsky, 1874) for specimens collected in Ensenada de La Paz, BCS, Mexico (Galicia-Nicolas et al. 2018; Mendoza 2019; Cardona-Gutiérrez 2021; Cruz-Escalona et al. 2021). However, these studies only assumed, without any proper taxonomic evaluation, that the individuals collected at Ensenada de La Paz belonged to this species reported in other regions of the world.

The goal of the present study is to describe the morphology of the ascidian (allegedly *D.stylifera*) collected in Ensenada de La Paz, BCS, Mexico, and discuss its taxonomic status as compared with previous descriptions of *D.stylifera* in other regions of the world.

## ﻿Materials and methods

**Figure 1.**
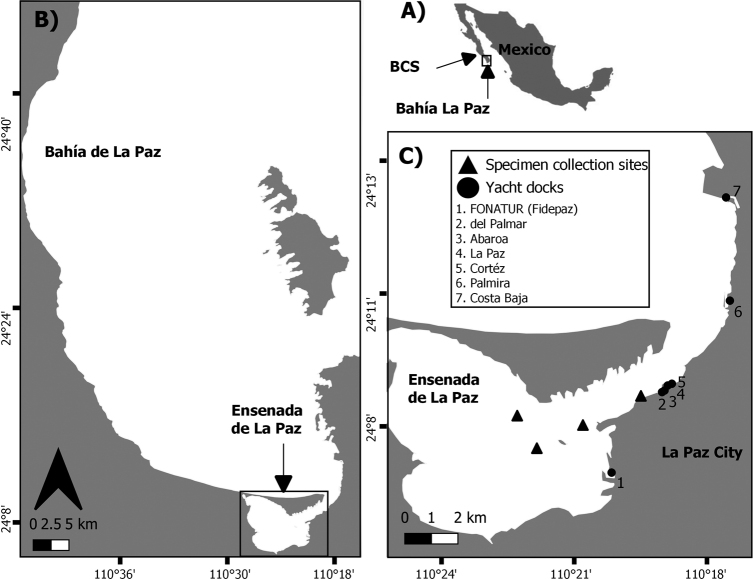
**A** area of study in Ensenada de La Paz located in the southern part of Bahía La Paz, Baja California Sur, Mexico **B** both bodies of water are located on the southwest coast of the Gulf of California, Mexico **C** sampling sites and potential sources of dispersal of tunicates (circles) [FONATUR (Fidepaz) (24°07'3"N, 110°20'47"W), del Palmar (24°09'09"N, 110°19'39"W), Abaroa (24°09'11"N, 110°19'36"W), La Paz (24°09'17"N, 110°19'31"W), Cortéz (24°09'19"N, 110°19'26"W), Palmira (24°10'58"N, 110°18'09"W) and Costa Baja (24°13'07"N, 110°18'12"W)].

The study area (Bahía de La Paz) is located southeast of Baja California Sur (24°07'05"N, 110°17'08"W and 24°80'85"N, 110°70'18"W) (Fig. 1A, B). The Ensenada de La Paz is separated from the Bahía de La Paz by a 12 km long (0.4–2.8 km wide) sandy bar known as El Mogote (Cruz-Orozco et al. 1989; Obeso-Nieblas et al. 2008) (Fig. 1A–C). The Ensenada has an area of ~ 45 km^2^ and a maximum depth of 10 m and is connected to the Bahía de La Paz through a shallow channel (< 10 m depth) approximately 1 km wide and 4 km long (Fig. 1C). The bottom of the cove ranges from sand to mud-silt (Espinoza 1977). The conurbation of La Paz city, with 300,000 people, has seven yacht docks (Fig. 1C), and a commercial harbor Pichilingue located at the entrance of Bahía de La Paz.

Colonies of the three different color morphs (white, orange, and purple) of Distapliacf.stylifera were collected with autonomous diving, using a spatula to extract the colonies from the substrate in four sampling sites of Ensenada de La Paz BCS (24°8'01"N, 110°23'26"W; 24°7'29"N, 110°22'25"W; 24°8'41"N, 110°21'52"W; 24°9'31"N, 110°20'16"W) during December 2017, June 2021, and October 2021, to investigate the identity of the species that caused the mass mortality event of pen shells *Atrinamaura* (Moreno-Dávila et al. 2021). The specimens were relaxed with menthol crystals for taxonomic examination. After observing live specimens with a stereoscope, some of the collected ascidians were fixed and preserved in 5% formalin for further morphological observation. Twelve colonies were preserved in 96% undenatured ethanol and the zooids were extracted to obtain sequences of the mitochondrial Cytochrome Oxidase I (COI) gene.

Morphological observations were carried out on relaxed and preserved material using a stereomicroscope with mounted digital camera. Staining was performed when necessary, using Masson’s Hemalum. These morphological observations were compared with relevant descriptions from the literature (see Discussion).

For molecular analyses, zooids from 12 ascidian colonies with white, orange, and purple coloration were dissected and their digestive tracts removed to minimize contamination. Their genomic DNA was extracted using the Qiagen DNeasy Blood & Tissue kit (Qiagen, Valencia, CA), following the manufacturer’s protocol. To amplify a fragment of the mitochondrial Cytochrome Oxidase I (COI) gene, different primer sets were used, either universal (Folmer et al. 1994) or specific for tunicates (Iannelli et al. 2007; Stefaniak et al. 2009; Brunetti et al. 2017; Salonna et al. 2021). We also tried a nested PCR strategy following Salonna et al. (2021): A first amplification was done with the primer pair dinF/Nux1R, and then a 1:100 dilution of the PCR product was used as template for the second PCR with the primer pair cat1F/ux1R. PCR amplification followed in 20 μl total reaction volume with 2.5 mM MgCl2, 0.3 mM dNTPs, 1X buffer, 0.3 µM of each primer, and 0.16 U of Taq DNA polymerase (Invitrogen, Inc., Carlsbad, CA). The PCR program consisted of an initial denaturing step at 95 °C for 5 min, 35 amplification cycles (denaturing at 95 °C for 1 min, annealing at 43 °C for 1 min and extension at 72 °C for 1 min), and a final extension at 72 °C for 5 min. Positive PCR products were purified and sequenced by Macrogen Inc. in forward and reverse strands.

### ﻿Description

#### ﻿ Distaplia stylifera

Taxon classificationAnimaliaAplousobranchiaClavelinidae

(Kowalevsky, 1874)

62A7978A-4C3A-5655-B2F5-74AC5D837FB5

Figs 2 , 3 , 4

Didemnium styliferum Kowalevsky, 1874: 443, pl. 30, figs 1–16.
Holozoa bursata Van Name, 1921: 366–368, figs 44–47.
Distaplia bursata – Van Name 1930: 456, fig. 31.
Distaplia mikropnoa – Hartmeyer 1919: 130.
Distaplia stylifera – Hartmeyer 1919: 135; Michaelsen 1930: 502; Van Name 1945: 147, fig. 71.
Polyclinum mikropnous – Sluiter 1909: 94, pl. 5, fig. 1.

##### Material examined.

CEAB.ASC.DIST–001: Ensenada de La Paz, Mexico; two purple colonies, one as epibiont on sea pen *A.maura* and one attached to a PVC pipe, 1–3 m depth, 03/Dec/2017. CEAB.ASC.DIST–002: Ensenada de La Paz, Mexico; two white colonies, one epibiont on *A.maura* and one attached to a buoy, 1–3 m depth, 03/Dec/2017. CEAB.ASC.DIST–003: Ensenada de La Paz, Mexico; three orange colonies epibiont on *A.maura*, on a rope and on a buoy, 1–3 m depth, 03/Dec/2017. CEAB.ASC.DIST–004: Ensenada de La Paz, Mexico; three orange colonies attached to wooden yacht docks, 0.5 m depth, 19/Jun/2021. CEAB.ASC.DIST–005: Ensenada de La Paz, Mexico; three purple colonies attached to wooden yacht docks, 0.5 m depth, 19/Jun/2021. CEAB.ASC.DIST–006: Ensenada de La Paz, Mexico; three white colonies on wooden yacht docks, 0.5 m depth, 19/Jun/2021. CEAB.ASC.DIST–007: Ensenada de La Paz, Mexico; eight orange colonies on wooden yacht docks, 0.5 m depth, 9/Jul/2021. All the colonies examined in the present study have been deposited in the Biological Collection of the Center of Advanced Studies of Blanes (CEAB) with voucher codes CEAB.ASC.DIST–001 to 007.

##### Morphological characters.

**Figure 2.**
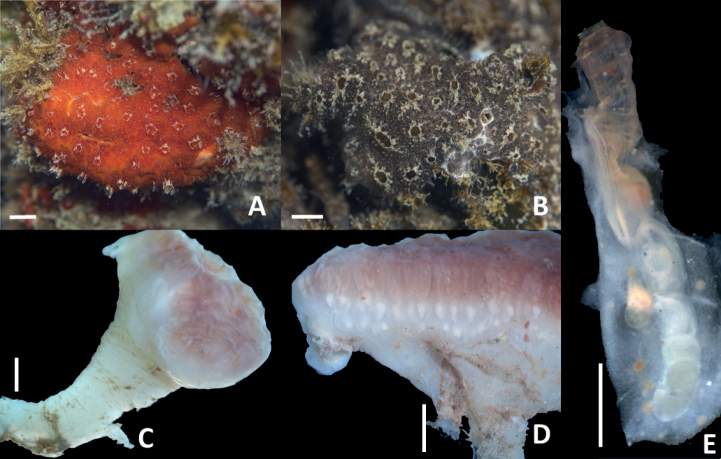
*Distapliastylifera***A** live orange colony **B** live purple colony **C, D** typical mushroom-shaped colonies **E** Zooid. Scales bars: 10 mm (**A, B**); 2.5 mm (**C, D**); 1 mm (**E**).

Colonies can be mushroom-like with orange color and white mottles marking the common cloacal apertures (Fig. 2A). Purple (Fig. 2B) or white colonies (not shown) can also be found. They are up to 2 cm in height and 2–2.5 cm in head (cormidium) diameter (Fig. 2C, D). The basal part of the colonies is less colored, and only the distal region had pigment after preservation. The tunic is firm and sometimes the stalks are branched so that different heads originate from the same base. Each head had one or several zooid systems, each with a central common cloacal aperture to where double rows of zooids converge. Small systems are made up of a simple circle of zooids. Colonies can also be cushion-shaped, spread over the substrate without a stalk.

**Figure 3.**
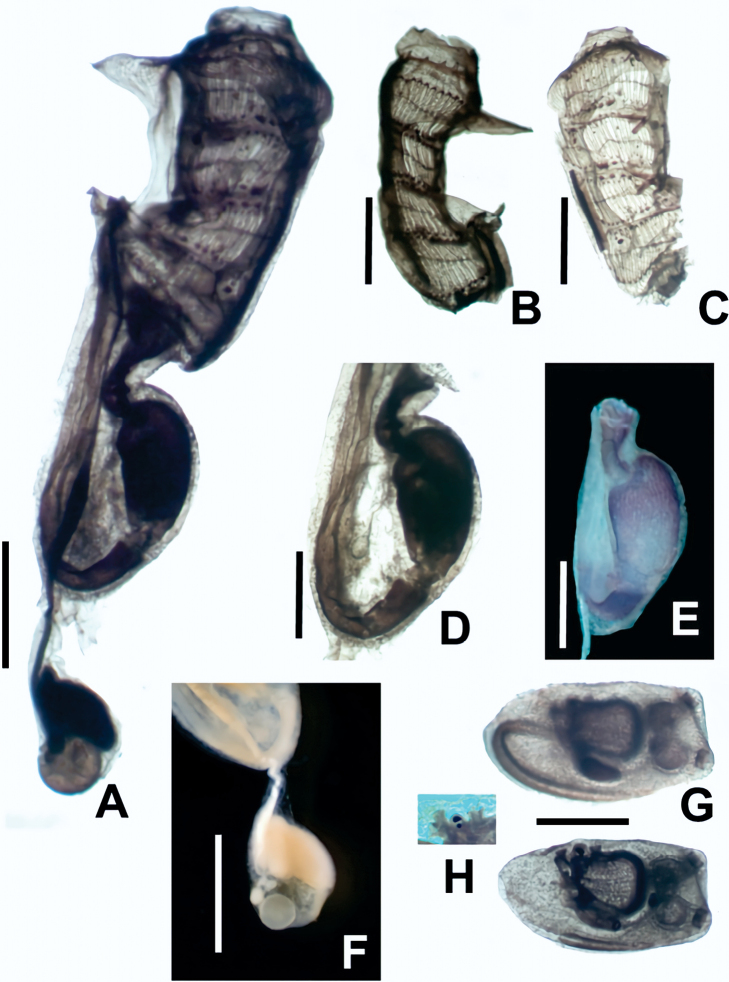
*Distapliastylifera***A** zooid (thorax and abdomen) **B** thorax **C** dissected thorax **D, E** stomach **F** gonads **G** larvae **H** enlargement of one larva showing two pigmented spots. Scales bars: 10 mm (**A**); 0.5 mm (**B, C, F, G**); 0.25 mm (**D, E**). All images (except **F**) correspond to stained zooids.

The zooids are up to 5 mm in length (excluding the gonadal sacs) (Fig. 2E). They are divided into thorax and abdomen, with two stalked sacs adhered. One sac, smaller than the abdomen and connected to its right posterior side (Fig. 3A), includes the gonads. The second sac contains embryos and larvae in incubation and can be longer than the zooid itself. It is attached by a thin peduncle joining the zooid at the posterior part of the pharynx, on the right-hand side close to the dorsal line (Fig. 2E).

**Figure 4.**
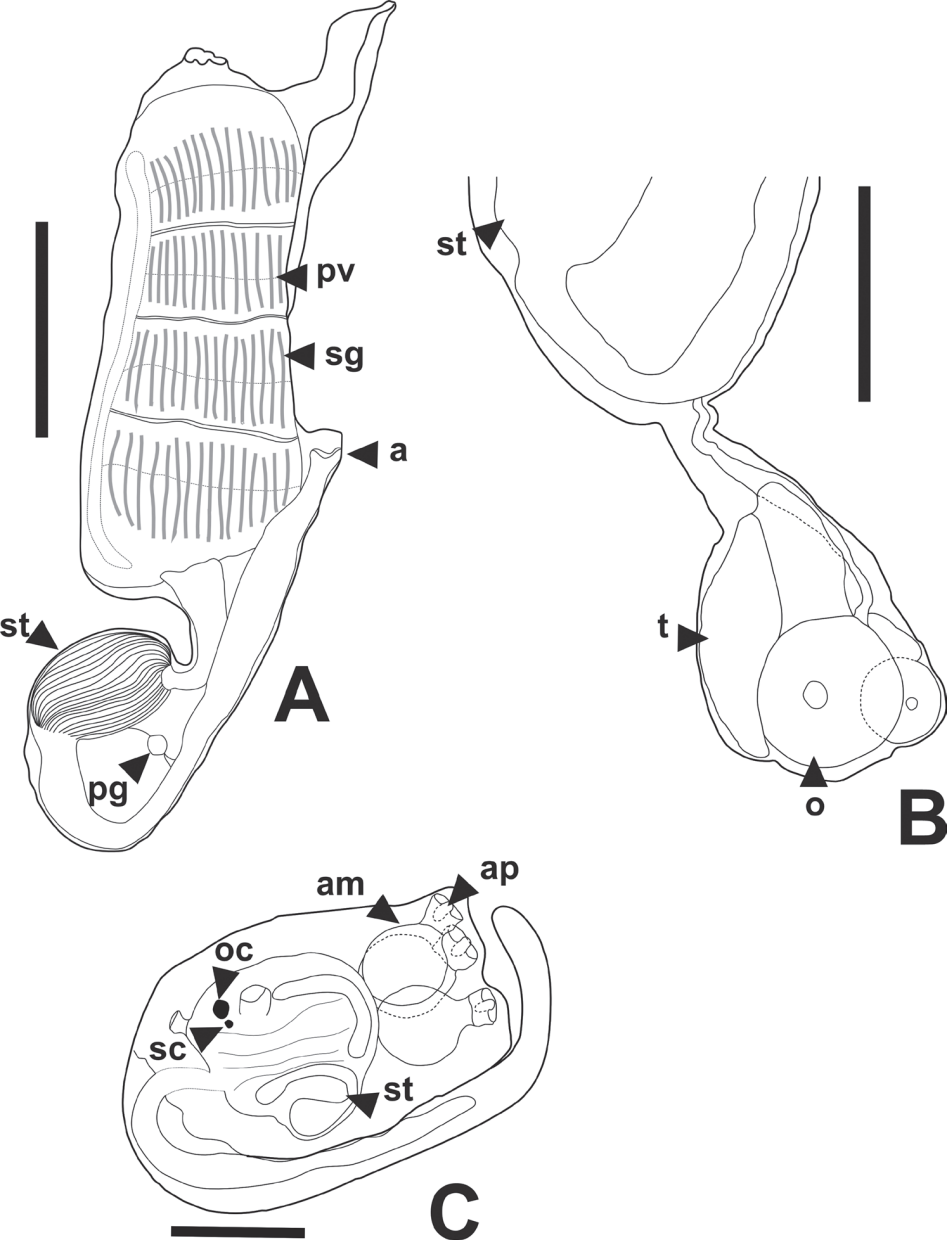
*Distapliastylifera***A** zooid (thorax and abdomen) **B** abdomen **C** larva. Abbreviatures: **a.** anus; **am.** ampullae; **ap.** adhesive papillae; **oc.** ocellus; **o.** oocyte; **pv.** parastigmatic vessels; **pg.** pyloric gland vesicle; **sc.** statocyte, **sg.** stigmata; **st.** stomach; **t.** testes. Scales bars: 1 cm (**A**); 0.5 mm (**B, C**).

The thorax has a smooth-rimmed oral siphon (or with six slight lobulations), with a large atrial aperture exposing most of the branchial sac. An atrial languet, often consisting of a wide flap-like lid with smooth or lobed margins, is placed at the top of the atrial aperture (Figs 3A, 4A). There are several transverse muscular bands across the atrial languet. Approximately 30 longitudinal muscular bands run over the thorax of each side. The thorax has ~ 14 simple oral tentacles. The pharynx has four stigmata rows clearly divided by parastigmatic vessels (Fig. 4A). All thoraces examined (except for those recently budded) had these parastigmatic vessels. The number of stigmata per half-row are typically between 18 or 19 (reaching 22 in larger zooids) in the first two rows and 15 or 16 (reaching 18) in the two posterior rows. There are three simple dorsal languets between rows, slightly displaced to the left-hand side (Fig. 3B, C).

The abdomen has an elongated and curved stomach. Its wall is marked by fine plications (> 20 per side) that, in section, are visible in both the outer and the inner surface of the wall (Figs 3D, E, 4B). The plications are longitudinal but can be interrupted or divided. There is a short post-stomach that connects to an enlarged mid-intestine located at the bottom of the gut loop. The distal intestine runs anteriorly and ends in a bilobed anus at the base of the atrial aperture. The pyloric gland features a vesicle or reservoir between the stomach and the intestine and continues anteriorly forming sinuous tubules over the intestine in front of the stomach (Figs 3D, E, 4A).

The gonads lie in a pedunculated sac, with one well-formed oocyte (sometimes a smaller second one) at the bottom and a cluster of five or six elongated or wedge-shaped testes placed vertically. The common sperm duct arises posteriorly from the cluster of testes, but turns anteriorly at its very beginning, without overlapping the oocytes (Figs 3F, 4B).

All *Distaplia* colonies examined had larvae incubating in long sacs that reach posteriorly deeper than the zooids themselves in the colonies. Usually, several larvae can be seen in the brooding sacs, containing up to two well-formed larvae plus three embryos. Larvae are ~ 1.3 mm length, and when fully developed their body become elongated, reaching up to 1.5 mm in trunk length. Larvae possess three adhesive papillae, two dorsal and one ventral, with a globular ampulla each in the stalks. The oozooid is well-formed, with four rows of stigmata already present and an incipient abdomen folded under the branchial sac (Figs 3H, 4C). The sensory vesicle contains two pigmented spots, a larger one (likely corresponding to the ocellus) and a smaller one (likely the otolith) just under it (Fig. 3G). The pigmented spots are very close and, given that the larvae are not completely transparent, the two pigmented spots can be easily taken as one.

##### Geographic distribution.

**Figure 5.**
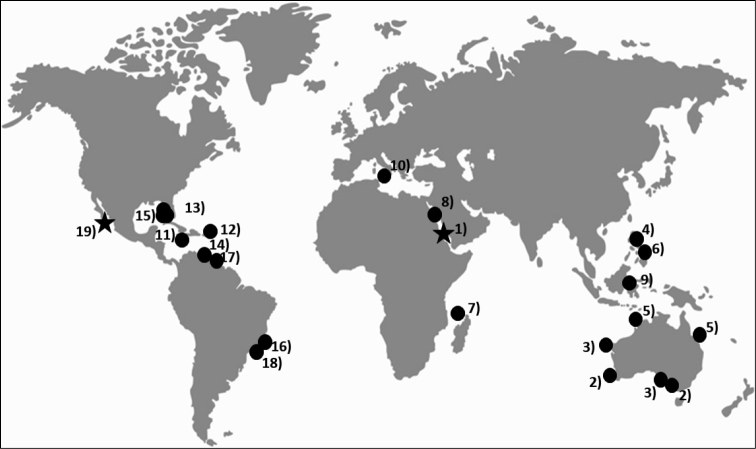
Sites of previous records of *Distapliastylifera*: 1) Red Sea, Kowalevsky 1874 (type locality). Indo-Pacific, 2) Brewin (1953). 3) Millar 1963 (1966); 4) Millar (1975); 5) Kott 1990 (2002); 6) Monniot and Monniot (2001); 7) Monniot (2012); 8) Shenkar (2012); 9) Lee et al. (2016). Mediterranean, 10) Pérès (1956). Western Atlantic Ocean, 11) Van Name (1921, 1945); 12) Monniot and Monniot (1984, Monniot 1988); 13) Cole and Lambert (2009); 14) Rocha et al. (2010); 15) Villalobos et al. (2017); 16) Rocha et al. (2011); 17) Streit et al. (2021); 18) Dias et al. (2013). Eastern Pacific Ocean, 19) present study. The type locality in the Red Sea and the record of the present study are indicated with stars.

*Distapliastylifera* was described in the Red Sea (Kowalevsky 1874) and has been reported in several areas of the Indo-Pacific. It has been found in Australia (e.g., Brewin 1953; Millar 1963, 1966; Kott 1990, 2002), Philippines (Millar 1975; Monniot and Monniot 2001), South China Sea (Lee et al. 2016), Madagascar (Monniot 2012), and (as *D.mikropnoa*, a debated synonymy, see Discussion) in Palau Islands (Tokioka 1955, 1967). There is a dubious report in the Mediterranean Sea (Pérès 1956). It is also known from the Western Atlantic coast from North Carolina to Jamaica (Van Name 1921, as *D.bursata*, Van Name 1945; Villalobos et al. 2017). It has been reported in several Caribbean locations (Monniot and Monniot 1984; Monniot 1988; Rocha et al. 2010; Streit et al. 2021), and there are recent reports further south in artificial substrates at Sao Paulo region, Brazil (Rocha et al. 2011; Dias et al. 2013). It therefore appears to have a wide circumtropical distribution (Fig. 5, but see discussion for potential taxonomic issues), although it has never been documented in the Eastern Pacific.

##### Molecular analyses.

Despite obtaining positive amplifications with all the primer pairs assayed, no sequence could be obtained that blasted with ascidian mitochondrial COI. Our sequences were closer to algae, mycoparasites or bacteria with an 80–83% similarity. These results may be due to the presence of mutations in the binding sites or contamination. However, we consider contamination unlikely given the care taken during the extraction of the samples.

## ﻿Discussion

The taxonomy of the genus *Distaplia* is mainly based on characters such as colony shape, arrangement of zooids in systems, presence or not of gonadal sac, stigmata per row, stomach shape and external surface, and muscle arrangement (Van Name 1945; Kott 1990; Monniot and Monniot 2001). According to these morphological characters, the specimens collected at Ensenada de La Paz between 2015 and 2017 reported in Moreno-Dávila et al. (2021) and in the present study match well with the detailed descriptions of *D.stylifera* given by Van Name (1945), Monniot (1988), and Monniot and Monniot (2001). The original description of Kowalevsky (1874) as *Didemniumstyliferum* lacks some key characters, as is often the case with old descriptions: the presence of parastigmatic vessels was not mentioned, and the holotype specimen was likely juvenile, with under-developed gonads. In addition, the colonies were lacking larvae.

However, the descriptions of *D.stylifera* reported in the literature are not entirely consistent in several morphological characteristics. As is common in widespread colonial ascidians, it is possible that worldwide reports of *D.stylifera* encompass a group of closely related species. One critical diagnostic morphological characteristic is the presence or absence of parastigmatic vessels. All the colonies collected at Ensenada de La Paz had parastigmatic vessels. Kott (1990) reported that all the specimens collected in Australia, except one colony, lacked parastigmatic vessels. Monniot (1988) reported them in material from the Caribbean but noted the absence of parastigmatic vessels in colonies collected from Madagascar (Monniot 2012). The absence of parastigmatic vessels has been pointed out as the main characteristic distinguishing *D.stylifera* from *D.mikropnoa* (Monniot and Monniot 2001), and Millar (1975) indicated that the variation in this character suggested that these species were synonymous. However, Kott (2002) refers to other differences distinguishing *D.mikropnoa* from *D.stylifera* such as the long double rows of zooids converging to the terminal common cloacal apertures, a long post-pyloric part of the gut loop, and the lack of a gastric reservoir. Another difference is the course of the gastro-intestinal ducts that does not cross from the stomach to the ascending limb of the gut loop but extends down the descending loop. She therefore concluded that both are valid biological species but have been confused in the literature. In particular, the reports of Tokioka (1955, 1967) of *D.mikropnoa* in Palau correspond to *D.stylifera* according to Kott (1990).

Several authors reported *D.stylifera* testis with as many as 15 oval follicles (Tokioka 1955, 1967; under the name *D.mikropnoa*Kott 1990). The specimens from Ensenada de La Paz had only five or six elongated follicles, in accordance with previous reports of *D.stylifera* (Van Name 1945; Monniot 1988; Monniot and Monniot 2001). Further, the sperm duct of *D.stylifera* is sometimes described as running posteriorly and making one or several loops over the oocytes before turning anteriorly (Kott 1990; Monniot 2012), while in other descriptions the sperm duct is straight (Van Name 1945; Monniot and Monniot 2001), as in our specimens. In addition, the gonadal sac can be pedunculated, as in our specimens, or can be almost flush with the abdomen, separated by a wide neck (Kott 1990; Monniot 2012). Millar (1975) reported a single pigmented spot (the ocellus) in the larva, while other authors describe two pigmented spots. The specimens of Ensenada de La Paz have both ocellus and otolith; although it is not easy to discern them as distinct, which can explain previous confusion in the number of pigmented spots.

We consider that these variable morphological characters (presence or absence of parastigmatic vessels, number and shape of testes and sperm duct, gonadal pouch stalked or not stalked) indicate that several species have been mixed under the taxonomic name *D.stylifera*, as suggested also by Dias et al. (2013). This taxonomic uncertainty, as well as the potential synonymy with the morphologically close *D.mikropnoa* species, can be potentially solved by further comparative morphological and genetic analyses of specimens from different regions of the world. Even keeping in mind these shortcomings, the binomen *D.stylifera* has been reported in tropical zones of the Indian Ocean, the western Pacific, and the western Atlantic. Thus, our finding in the eastern Pacific represents an important range expansion of the species.

Unfortunately, we could not obtain sequences of our colonies. There are no sequences of *Distapliastylifera* available in GenBank and BOLD public databases, either. COI data for the single well-represented species of *Distaplia* in these databases, *D.bermudensis* Van Name, 1902, revealed divergences ~ 14–20% for different morphotypes and distribution areas. This indicates either high intraspecific variability or the existence of multiple species under *D.bermudensis* binomen, as suggested by some authors (Evans et al. 2021; Virgili et al. 2022). The high variability found in the congener *D.bermudensis* suggests that shedding light on the relationships of *D.stylifera* will require extensive sampling of the main areas where the species has been recorded to settle whether it is a single polymorphic biological species with circumtropical biogeographic distribution or a group of related species with distinct (perhaps overlapping) biogeographic distribution patterns.

Interestingly, the species (or some members of the species complex if it proves to be so) has been likely introduced in some parts of its distribution. In western Atlantic it is found from North Carolina to Brazil. Van Name (1921), under the name *D.bursata*, records it in Florida and Jamaica samples collected as early as 1884. However, in North Carolina and in the Caribbean Sea it is mostly found in lagoons and on artificial structures, typical entry points for introduced species (Rocha et al. 2010; Dias et al. 2013; Villalobos et al. 2017; Streit et al. 2021). Its status in the Caribbean has been defined either as cryptogenic (Streit et al. 2021) or as introduced species (Rocha et al. 2010). If the introduced status is eventually confirmed in that area, this species poses a worrisome threat due to its ability to rapidly colonize surfaces and displace other benthic species (Rocha et al. 2010). The species seems to be expanding southwards and was present since the 2000’s in the Sao Paulo region of Brazil, where it was found only on artificial substrates (Dias et al. 2013).

Albeit taxonomic uncertainty impedes sound interpretation of biogeographical patterns, given the mass mortality event of pen shells in Ensenada de La Paz caused by *D.stylifera*, it can behave as a typical impactful invasive species. This fact, coupled with a likely previous introduction history in the Western Atlantic and the lack of reports in the Eastern Pacific, strongly suggests that it is a new arrival to the Gulf of California. The species was first noticed by NOS (2015) in June 2015, in spite of periodic monitoring of the area since 2011 for evaluation of the bivalve stocks. It is unclear whether the population detected in this region was introduced from western Pacific populations, or from Atlantic populations via the Panama Channel. Ship traffic seems the most likely introduction vector, and secondary spread by small craft to harbors in the vicinity of the Ensenada de la Paz can account for its arrival there. The origin, the number of introduction events and the existence or not of bottlenecks during the introduction can only be ascertained with detailed genetic studies of worldwide populations. For the time being, constant monitoring and eradication actions seem the only feasible measures to control its growth and to avoid further expansion (Valéry et al. 2008, 2009; Warren 2021).

## Supplementary Material

XML Treatment for Distaplia stylifera
